# Improved Drought Monitoring Index Using GNSS-Derived Precipitable Water Vapor over the Loess Plateau Area

**DOI:** 10.3390/s19245566

**Published:** 2019-12-16

**Authors:** Qingzhi Zhao, Xiongwei Ma, Wanqiang Yao, Yang Liu, Zheng Du, Pengfei Yang, Yibin Yao

**Affiliations:** 1 College of Geomatics, Xi’an University of Science and Technology, Xi’an 710054, China; 17210062025@stu.xust.edu.cn (X.M.); sxywq@xust.edu.cn (W.Y.); 18210062021@stu.xust.edu.cn (Y.L.); 19210061041@stu.xust.edu.cn (Z.D.); 19210061031@stu.xust.edu.cn (P.Y.); 2 School of Geodesy and Geomatics, Wuhan University, Wuhan 430079, China; ybyao@whu.edu.cn

**Keywords:** SPEI, temperature, TH model, RTH model, PWV

## Abstract

Standardized precipitation evapotranspiration index (SPEI) is an acknowledged drought monitoring index, and the evapotranspiration (ET) used to calculated SPEI is obtained based on the Thornthwaite (TH) model. However, the SPEI calculated based on the TH model is overestimated globally, whereas the more accurate ET derived from the Penman–Monteith (PM) model recommended by the Food and Agriculture Organization of the United Nations is unavailable due to the lack of a large amount of meteorological data at most places. Therefore, how to improve the accuracy of ET calculated by the TH model becomes the focus of this study. Here, a revised TH (RTH) model is proposed using the temperature (T) and precipitable water vapor (PWV) data. The T and PWV data are derived from the reanalysis data and the global navigation satellite system (GNSS) observation, respectively. The initial value of ET for the RTH model is calculated based on the TH model, and the time series of ET residual between the TH and PM models is then obtained. Analyzed results reveal that ET residual is highly correlated with PWV and T, and the correlate coefficient between PWV and ET is −0.66, while that between T and ET for cases of T larger or less than 0 °C are −0.54 and 0.59, respectively. Therefore, a linear model between ET residual and PWV/T is established, and the ET value of the RTH model can be obtained by combining the TH-derived ET and estimated ET residual. Finally, the SPEI calculated based on the RTH model can be obtained and compared with that derived using PM and TH models. Result in the Loess Plateau (LP) region reveals the good performance of the RTH-based SPEI when compared with the TH-based SPEI over the period of 1979–2016. A case analysis in April 2013 over the LP region also indicates the superiority of the RTH-based SPEI at 88 meteorological and 31 GNSS stations when the PM-based SPEI is considered as the reference.

## 1. Introduction

Climate change in drought/humidity is closely related to human activity and production and has an important impact on socioeconomic and regional ecological development [1,2]. Some drought indexes, such as Palmer drought severity index (PDSI), standardized precipitation index (SPI), and standardized precipitation evapotranspiration index (SPEI), have been proposed to monitor and quantify drought [1,3,4,5]. Drought is a water deficit phenomenon caused by low precipitation in a given period of time [4]. Under the background of global change characterized by warming, changes in temperature and precipitation and other factors affect the balance of surface water revenue and expenditure, which ultimately changes the surface drought/humidity situation [6,7].

At present, three types of meteorological drought indices are commonly used for drought monitoring. First, the PDSI is calculated based on soil water balance [3]. This index has been constantly revised and improved since its proposal. However, the inherent limitations of this index still exist; such defects include having a scale of drought assessment of 9–12 months and being affected by the index of the previous four years due to its autoregressive characteristics [8]. Second, the SPI can study drought at different spatial scales and has good comparability with PDSI in 6–12 months [4]. Although this index has been used in some drought studies [8,9,10,11,12], it only considers the contribution of precipitation. Third, the SPEI synthesizes the advantages of PDSI in evaporation and SPI on multiple time scales [5]. This index is commonly used in drought research worldwide [13,14,15].

Evapotranspiration (ET) is one of the most important parameters in SPEI calculation, which can be calculated using various formulas. In calculating SPEI, ET is obtained based on the Thornthwaite (TH) model [16], which only requires temperature because global temperature data are easy to obtain. However, aerodynamic and radiation terms are neglected when calculating ET based on the TH formula, and the ET cannot be calculated when the average monthly temperature in winter is less than 0 °C. At present, the Penman–Monteith (PM) model is the standard calculation method of ET recommended by the Food and Agriculture Organization (FAO) of the United Nations. The American Society of Civil Engineers analyzed the accuracy of 20 related formulas and compared them with the measured data of a lysimeter under 11 climatic conditions distributed around the world. The compared result reveals that the PM formula was proven to be close to the measured value in arid and wet areas [17]. Sheffield et al. found that the TH model overestimated the values of drought monitoring index in global trends over the past 60 years [18]. Although more accurate ET values can be obtained using the PM model, this model requires many meteorological parameters. Generally, the meteorological data used in the PM model cannot be obtained, and accurate ET is unavailable in most areas of the world. Therefore, a more accurate model must be established to calculate ET with less meteorological data.

With the emerging of global navigation satellite system (GNSS) meteorology [19], the high-precision precipitable water vapor (PWV) can be obtained with the root mean square (RMS) value of 1–3 mm [20,21,22,23,24]. In recent years, PWV has been applied to various disaster events, such as extreme rainfall [25,26], floods [25,27], and hurricanes [28]. In addition, the relationship between GNSS-derived PWV and drought has also been investigated. For example, the drought in the Yunnan Province of China can be detected based on the abnormal trend of PWV and vertical crustal deformation [29]. Wang et al. found that the nonlinear trend of PWV can be used to monitor drought and flood disasters in Australia [30]. The GNSS-derived PWV is used in this paper to establish its relationship with ET.

In this paper, a revised TH (RTH) model is developed by introducing the PWV. The Loess Plateau (LP) area is in the arid and semi-arid areas of northern China. This area is severely affected by extreme climatic events, such as drought and extreme precipitation, which makes it an ideal place for drought monitoring [31]. Therefore, the LP region is taken as the study area in this work, in which 88 meteorological stations and 31 GNSS stations are selected for the experiment. The ET residual between PM and TH models is initially obtained, and the relationships between ET residual and PWV/temperature are then further analyzed. Therefore, a revised TH (RTH) model is established by combining the TH-derived ET and the ET residual estimated based on the residual model. Finally, the RTH-based SPEI can be calculated and compared with that from TH- and PM-based SPEI. This paper is organized as follows: Section 2 describes the data used and the methods; the Validation experiment is performed, and corresponding results and discussion are presented in Section 3; the conclusion is summarized in Section 4.

## 2. Data and Methods

### 2.1. Study Area

Located on the second step of China, LP is a complete loess type and typical area of geomorphological development in the world, and its average elevation is approximately 1000 m. From northwest to southeast, the maximum height difference is approximately 2000 m [32]. The climate of the LP belongs to the typical continental monsoon climate, which is mainly affected by the polar dry and cold airflow in winter and spring, and the climate is cold and windy. In summer and autumn, the climate is mainly affected by the high western Pacific subtropics and the low pressure in the Indian Ocean, which is hot and rainy [33]. The annual average precipitation is approximately 466 mm, with evident spatial variation. The annual precipitation increases from northwest to southeast, with rainfall of 200–750 mm, and 65% of the precipitation distributes from June to September. The estimated annual potential ET is considerably higher than that of precipitation, ranging from 865 mm to 1274 mm. The northwest region is a typical semi-arid region, which makes it a typical region for drought monitoring [33].

### 2.2. Retrieval of GNSS and ERA-Interim PWV

PWV can be obtained from some techniques, such as radio sounding, remote sensing, and lidar but with corresponding shortcomings such as low temporal-spatial resolutions, high cost, and low precision [34]. GNSS technique can also retrieve PWV with the advantages of low cost, all-weather conditions, and high precision. However, zenith total delay (ZTD) is first obtained before calculating the PWV using the GNSS technique. The ZTD is an average value by projecting some values of slant path with different azimuths and elevation angles into a vertical direction, and the accuracy of GNSS-derived ZTD is approximately 4 mm [35]. When the radio signal crosses the troposphere, the corresponding delay will be generated, which is influenced by the atmospheric refraction effect [36]. In this study, the ZTD data is calculated using the GNSS observations derived from Crustal Movement Observation Network of China (CMONOC) based on GAMIT/GLOBK software (http://www-gpsg.mit.edu/~simon/gtgk/down.htm). ZTD consists of zenith hydrostatic delay (ZHD) and zenith wet delay (ZWD), where the ZHD can be precisely calculated based on an empirical model, such as the Saastamoinen model [37]. Therefore, the ZWD parameter, which is used to calculate PWV, can be obtained by extracting ZHD from ZTD. The specific formula for calculating PWV from ZWD is as follows [38]:

$$\mathrm{PWV}=\frac{10^{5}}{{(k}_{3}{/T}_{m}+k_{2}^{\prime }{)R}_{v}}\mathrm{ZWD}$$

where $T_{m}$ is the water-vapor-weighted atmospheric mean temperature, which can be calculated by the empirical model based on the observed temperature [39,40]. $k_{2}^{\prime }$, $k_{3}$ and R are the physical constants with values of 16.25 K/hPa, 3.776 × 10^5^ K^2^/hPa, and 461.495 J/K/kg, respectively.

The PWV value of the meteorological stations is interpolated using the PWV data of the four surrounding grid points. This data is from the fourth-generation atmospheric reanalysis of the global climate (ECMWF ERA-Interim), which covers rich data from 1979 to the present. ERA-Interim provides PWV, pressure, temperature, and other meteorological variables at grid points with different horizontal resolution (0.125° × 0.125° to 3° × 3°) globally. In this study, the monthly PWV values from the ERA-Interim reanalysis data with the spatial resolution of 0.25° × 0.25° were selected. The PWV values at meteorological stations are obtained based on the bilinear interpolation method using the above data.

### 2.3. Meteorological Data

**Figure 1:**
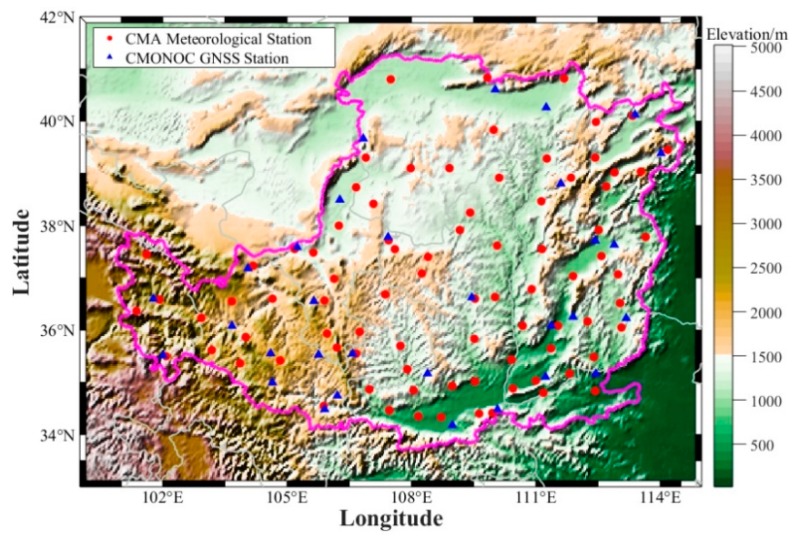
Geographic distribution of selected global navigation satellite system (GNSS) and meteorological stations over the Loess Plateau (LP) region.

Daily meteorological data of 88 stations that are evenly distributed in the LP region over the period of 1979–2016 were collected (Figure 1), which include highest and lowest temperature, precipitation, relative humidity, sunshine duration, and 2 m wind speed. These data are downloaded from the China Meteorological Sharing Network (http://data.cma.cn/) and have been rigorously checked by CMA’s staff. The work includes checking and marking the wrong and missing data, as well as supplementing those data before release. The monthly corresponding data were obtained using the daily meteorological data. The monthly temperature and precipitation of the Yang Kun dataset derived from the Qinghai–Tibet Plateau Scientific Data Center (http://www.tpedatabase.cn/portal/MetaDataList.jsp) were also used to interpolate the corresponding meteorological data at GNSS stations [41]. The specific process of generating such a dataset can be found in [41] and [42]. The temperature and precipitation are interpolated using the corresponding data of the four surrounding grid points derived from the Yang Kun dataset to calculate the SPEI value at selected GNSS stations.

### 2.4. Theory of ET and SPEI Calculation

ET is calculated based on the Thornthwaite (TH) and Penman-Monteith (PM) models, respectively, in this paper. TH model is widely used because it is easy to use and only needs monthly average temperature and latitude [43,44]. However, this model does not consider the influence of wind speed, air humidity, and other factors, and the value of ET is usually underestimated. Based on the energy balance equation and water vapor diffusion theory, the PM model not only considers the physiological characteristics of crops but also considers the changes in aerodynamic parameters. The calculated ET value based on the PM model is accurate. However, this model requires many meteorological parameters, such as the highest and lowest daily temperature, relative humidity, sunshine hours, and 2 m wind [17]. The specific processes of calculation ET based on TH and PM model have been given in Appendix A.

SPEI is an acknowledged good index for drought monitoring because it considers the superiority of PDSI and SPI. This index includes the temperature variability of PDSI and the multiscalar characteristic of SPI. Therefore, this index is suitable for drought monitoring with the rapidly changing climate scenarios. Before the calculation of SPEI, the difference between monthly precipitation and ST is first obtained, and then accumulating this difference in different time scales. Finally, the SPEI value can be calculated by standardized the accumulated difference. The specific calculation process has been given in Appendix B.

### 2.5. RTH Model

In this paper, the revised TH (RTH) model can be established following the three key steps:

1. Calculating the ET residual between TH and PM model:
  (1) $$V_{\mathrm{ET}}=\mathrm{PM}-\mathrm{TH}$$
2. Analyzing the time series of ET residual and fitting the ET residual model using the GNSS-derived PWV and temperature:
  (2) $$V_{\mathrm{ET}}=f(\mathrm{PWV},T)$$
3. Obtaining the initial ET value ${\mathrm{ET}}_{0}$ using TH model and establishing the ETH model using the ET residual:
  (3) $${\mathrm{ET}}_{\mathrm{RTH}}={\mathrm{ET}}_{0}+V_{\mathrm{ET}}$$

## 3. Experimental Results and Discussion

### 3.1. Missing Data Interpolation

**Figure 2:**
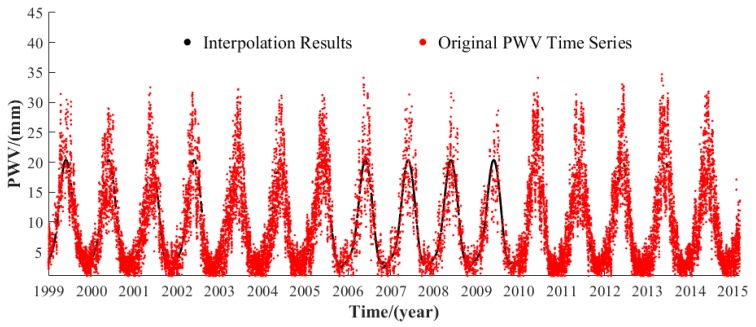
Interpolated time series of precipitable water vapor (PWV) at XNIN Station over the period of 1999–2015.

The GNSS stations from the CMONOC are selected, where the GNSS-derived PWV is obtained following the method proposed by [45]. A total of 31 GNSS stations exist over the LP region (Figure 1), and the average data missing rate at those stations is 13.8%. Therefore, an interpolated method referred to as SSAM (singular spectrum analysis for missing data) proposed by Wang et al. was used to interpolate the missing data at each GNSS station, and this method works well in interpolating the missing data of long-term PWV time series [46]. Figure 2 shows an example of the interpolated time series of the PWV time series at XNIN Station (36.6006° N, 101.7743° E) over the period of 1999–2015.

### 3.2. Comparison of ET Derived from Different Models

**Figure 3:**
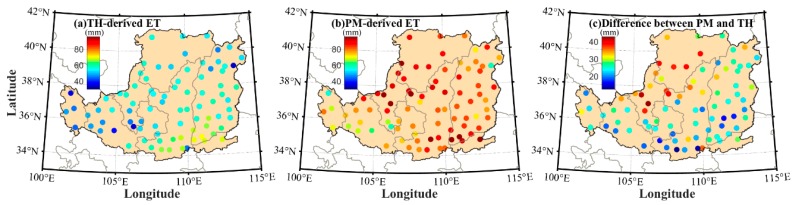
Average evapotranspiration (ET) values at 88 meteorological stations calculated based on Penman–Monteith (PM) and Thornthwaite (TH) models over the period of 1979–2016.

Figure 3a,b presents the calculated average ET values at 88 meteorological stations using TH and PM models over the period of 1979–2016. It can be observed that the accuracy of TH-derived ET values is poor at some stations when the PM-derived ET values are regarded as the reference. In addition, the ET differences of selected stations between the PM and TH models have been presented in the LP area. It can be observed that the maximum ET difference is almost 45 mm while the minimum ET difference is approximately 27 mm. Such a phenomenon demonstrates the necessity of establishing the improved model to correct the ET value.

**Figure 4:**
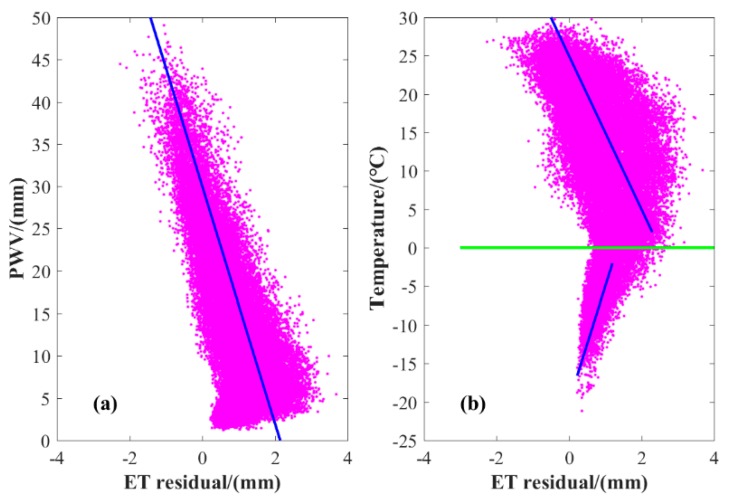
Relationships between ET residual and (**a**) precipitable water vapor (PWV) / (**b**) temperature (T) over the LP region, respectively.

Here, the ET residual between PM and TH models are obtained at 83 meteorological stations, and the relationships between ET residual and PWV/T are presented in Figure 4. It can be concluded that the ET residual has an evident negative linear relationship with PWV and T when T > 0 °C, whereas a positive linear relationship exists between ET residual and T when T < 0 °C. The different phenomena presented between T and ET residual for cases of T > 0 °C and T < 0 °C is mainly related to the 0 values of ET when T < 0 °C (Appendix A). The relationship between ET residual and altitude of meteorological stations is also analyzed, and no evident relationship exists. Therefore, a linear model between ET residual and PWV/T is established and expressed as follows:

(4) $$V_{\mathrm{ET}}=\left\{ \begin{array}{l} 56.6205-2.9494\cdot \mathrm{PWV}+1.1836\cdot T\;\;\;\;T>0\\ 39.4550-0.3899\cdot \mathrm{PWV}+1.854\cdot T\;\;\;\;\;\;T<=0 \end{array}\right.$$

### 3.3. Validation of the RTH Model

**Figure 5:**
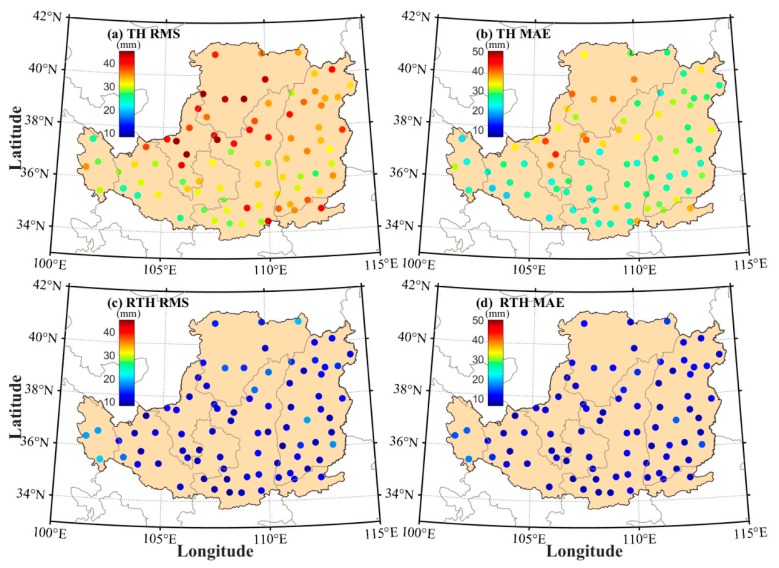
Comparisons of root mean square (RMS) and mean absolute error (MAE) of ET residual between TH and a revised Thornthwaite (RTH) models at 88 stations over the period of 2015–2016 when the ET derived from PM model is regarded as reference.

**Figure 6:**
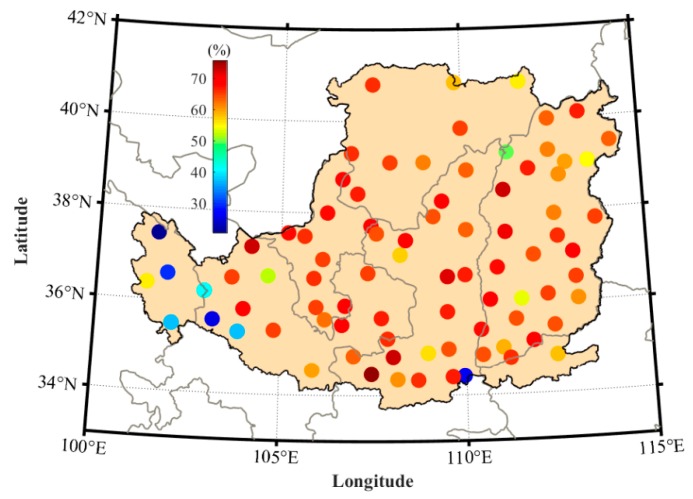
Average RMS improvement rate of RTH model compared with the TH model in the LP region over the period of 2015–2016.

To validate the proposed RTH model using the PWV and T over the LP region, only the meteorological data over the period of 1979–2014 were used to fit the coefficients of the ET residual model, whereas the data over the period of 2015–2016 were used to perform the validation experiment at 88 meteorological stations. Here, the ET values calculated by the PM model are regarded as reference. Figure 5 presents the comparisons of RMS and mean absolute error (MAE) of the ET residual between PM and TH/RTH models at 88 stations. It can be found that the values of RMS and MAE of the RTH model are smaller than those from the TH model at all stations when the values derived from the PM model are considered as reference. The statistical result reveals that the RMS and MAE derived from the RTH model are 12.3 and 10.2 mm, respectively, which are smaller than that from the TH model (34.2 and 29.2 mm, respectively). The RMS improvement rate of the RTH model is also calculated when compared with the TH model, as shown in Figure 6. It can be observed that the RMS improvement rate at different stations ranges from 22% to 75%, and the statistical result reveals that the average improvement rate of the RTH model is approximately 61.6%.

**Figure 7:**
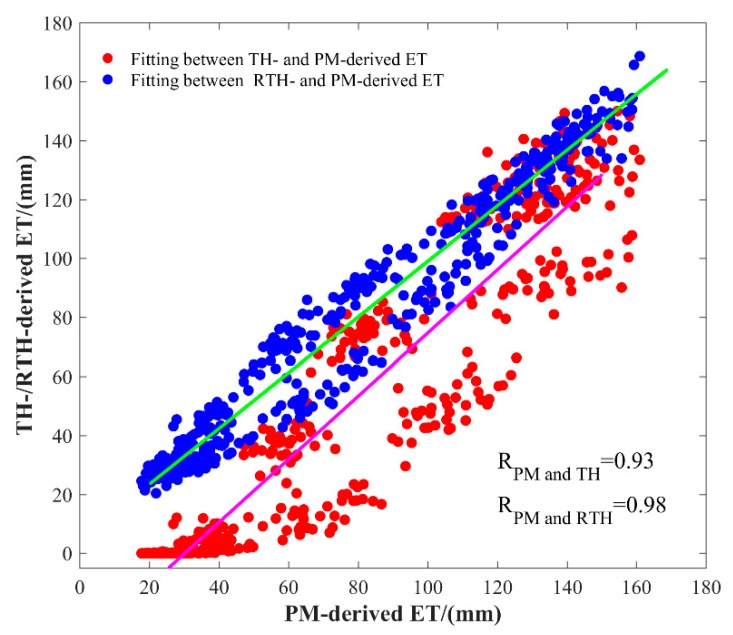
Scatter plot of monthly ET values calculated by TH, RTH, and PM models established in the LP region over the period of 2015–2016.

**Figure 8:**
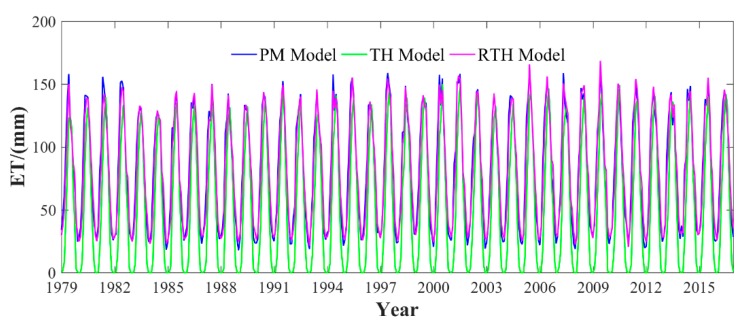
Long-term time series of average ET calculated using different methods in the LP region over the period of 1979–2016.

To further evaluate the complete accuracy of the established RTH model over the LP region, the scatter plot of monthly ET values calculated by TH, RTH, and PM models is shown in Figure 7. It can be observed that the RTH-derived ET has a better agreement with PM-derived ET when compared with that from TH-derived ET. The correlation coefficient between PM- and RTH-derived ET is 0.98, whereas the value between PM- and PH-derived ET is 0.93. The long-term time series of average ET calculated using different methods over the period of 1979–2016 in the LP region is shown in Figure 8. The time series of ET derived from the RTH model agrees well with that derived from the PM model, whereas the value of the TH model is relatively poor, especially for those times with low ET values, where the ET differences between PM and TH models are large. However, the RTH model established in this study offers an improvement for those values well, which is also the advantage of the RTH model.

### 3.4. Evaluation of RTH-Based SPEI at Meteorological Stations

After the ET value is obtained using the RTH model, the final SPEI can be calculated, which is called RTH-based SPEI in this study. In addition, the ET value is also calculated based on the PM and TH models. Therefore, the SPEI can also be calculated by such models, which is called PM- and TH-based SPEI. The quality of the RTH-based SPEI is the key to determining whether it can be further used. Therefore, the accuracy of RTH-based SPEI is initially evaluated and compared with TH-based SPEI when the PM-based SPEI is considered as reference.

**Figure 9:**
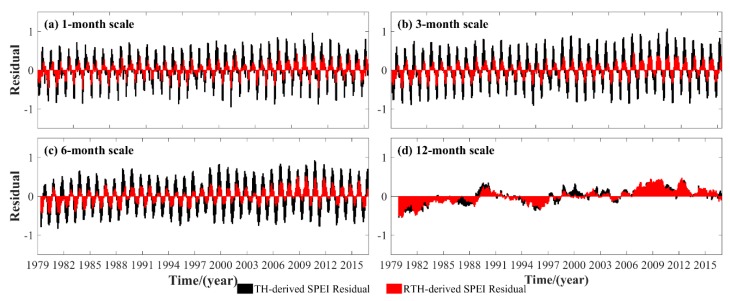
Long-term time series of average difference between RTH–PM- and TH–PM-based SPEI under multi-month scales at 88 meteorological stations over the period of 1979–2016.

The RTH-based SPEI is initially compared with TH-based SPEI at 88 GNSS stations over the period of 1979–2016, and the average SPEI differences between RTH–PM and TH–PM under 1-, 3-, 6-, and 12-month scales are presented in Figure 9. Those multi-month scales are selected and compared because they correspond to the monthly, seasonal, semi-annual, and annual scales. It can be observed from Figure 9 that the SPEI differences of RTH–PM are smaller than those from TH–PM, especially for the 1-, 3-, and 6-month scales, which further indicates the good performance of the RTH-based SPEI at those month scales.

**Figure 10:**
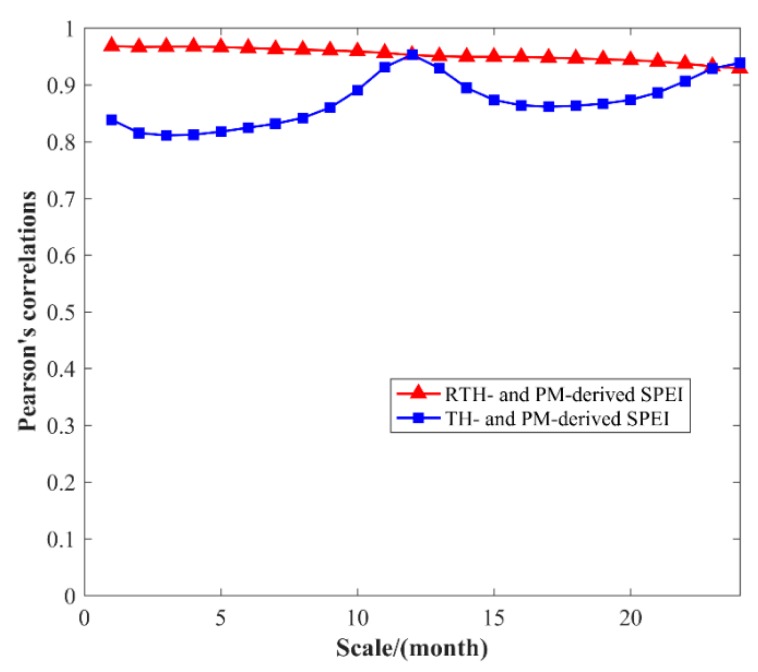
Pearson’s correlations of TH–PM- and RTH–PM-based SPEI under different multi-month scales.

Pearson’s correlation is then introduced in comparison to further analyze the relationship between SPEI calculated based on different models at different multi-month scales. Figure 10 shows the Pearson’s correlations of SPEI calculated based on different models under different multi-month scales from 1 to 24. The correlation between RTH- and PM-based SPEI is high at different month scales, especially for the 12-, 23-, and 24-month scales. Such a result conforms to the conclusion derived from Figure 9. In addition, it can be observed that the correlation values between TH-PM and RTH-PM are similar at 12-, 23-, and 24-month scales. This is because the monthly climatic water balance is standardized during the process of calculating SPEI (Appendix B). In those three scales, the changing trend of accumulated climatic water balances derived from TH and RTH models are both similar to those from the PM model. However, the values of the climatic water balance derived from the RTH model are closer to that from the PM model when compared to the TH model.

**Figure 11:**
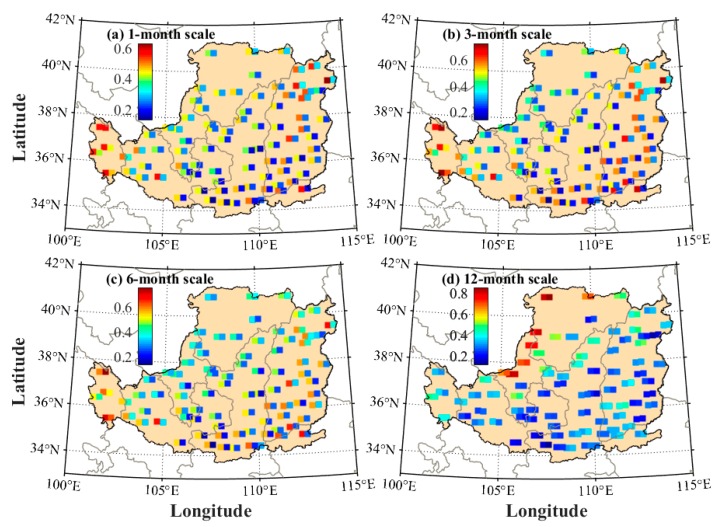
RMS comparison of SPEI difference between TH–PM and RTH–PM at each meteorological station over the period of 1979–2016, where the left/right squares at each station refer to the RMS derived from TH- and RTH-based SPEI, respectively.

**Figure 12:**
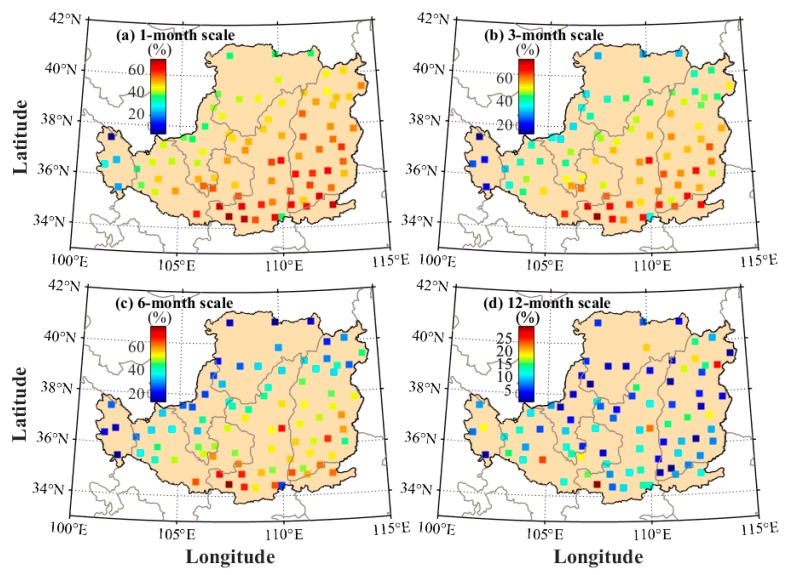
Average RMS improvement rate of RTH-based SPEI compared with TH-based SPEI in the LP region under different month scales.

The comparison of SPEI calculated using different models is also performed at each meteorological station under multi-month scales. The RMS values are presented in Figure 11. It can be observed that the RMS values of RTH-based SPEI are smaller than those of TH-based SPEI at all stations. Table 1 shows the statistical result of the average RMS and MAE of TH- and RTH-based SPEI when compared with the PM-based SPEI. The result shows that good performance can be obtained for the RTH-based SPEI. Finally, the average RMS improvement rate of the RTH-based SPEI compared with the TH-based SPEI is analyzed and presented in Figure 12 when the PM-based SPEI is considered as reference. It can be concluded that the accuracy of SPEI is improved to different degrees at different stations. The statistical result reveals that the average improvement rate of the RMS derived from the RTH-based SPEI is approximately 48.0%, 49.3%, 43.2%, and 9.1% under 1-, 3-, 6-, and 12-month scales in the LP region, respectively.

### 3.5. Case of Spatial Analysis of RTH-Based SPEI

**Figure 13:**
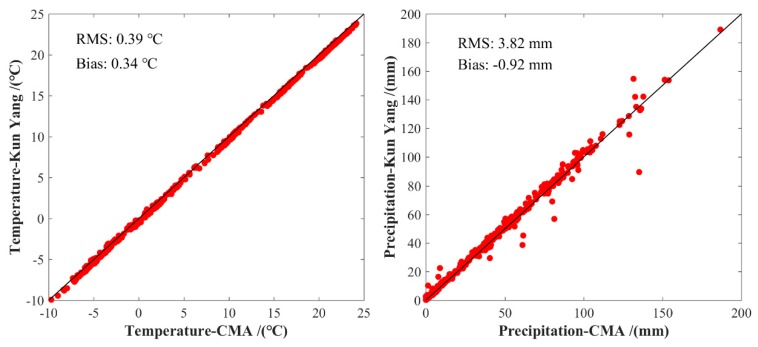
Scatter plots of temperature and precipitation in the LP region over the period of 1979–2016.

**Figure 14:**
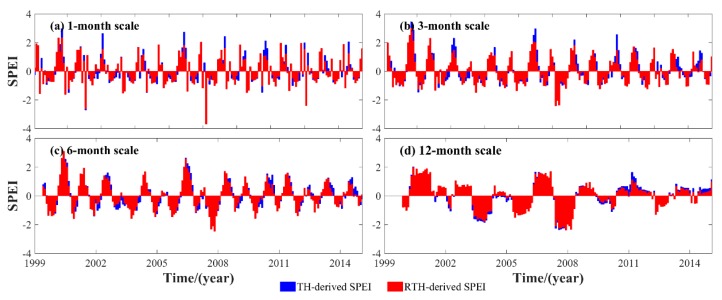
Comparison of TH- and RTH-based SPEI at XNIN Station over the period of 1999–2014 under different month scales.

The temperature and precipitation derived from the Yang Kun dataset is initially validated using the corresponding data derived from CMA at 88 meteorological stations. Figure 13 shows the scatter plots of temperature and precipitation over the period of 1979–2016 in the LP region. It can be observed that the temperature derived from the Yang Kun dataset agrees well with that from CMA, whereas the precipitation is relatively poor with that from CMA. The statistical result reveals that the RMS and bias of temperature and precipitation from the Yang Kun dataset are 3.85/−0.92 °C and 0.39/−0.34 mm, respectively. Therefore, the RTH-based SPEI can be calculated at GNSS stations using the corresponding data from the Yang Kun dataset and GNSS-derived PWV. Figure 14 presents an example of a comparison of the RTH- and TH-based SPEI at XNIN Station over the period of 1999–2014, where the PM-based SPEI cannot be calculated due to lack of corresponding data.

**Figure 15:**
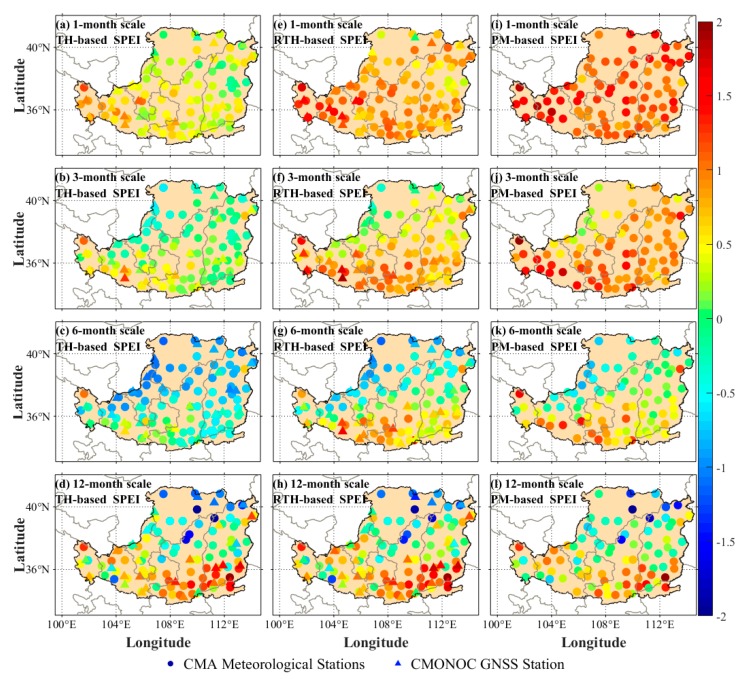
Comparison of SPEI calculated using different models at GNSS and meteorological stations in the LP region in April 2013 under multi-month scales, where the first, second, and third columns are the SPEI calculated based on TH, RTH, and PM models under different month scales.

According to the recordings of historical drought data of China meteorological network (https://cmdp.ncc-cma.net/cn/index.htm), a large surface drought occurred in the LP region in April 2013. Therefore, this month is selected to analyze the SPEI calculated based on the RTH model. Figure 15 shows the comparison result of the SPEI calculated using different models at GNSS and meteorological stations in April 2013. Due to the lack of corresponding meteorological data at GNSS stations, the PM-based SPEI cannot be obtained at the GNSS stations, which is also the disadvantage of the PM-based SPEI. It can be observed from Figure 15 that the RTH-based SPEI is superior to TH-based SPEI under different month scales when the PM-based SPEI is considered as reference, especially in 3-, 6-, and 9-month scales. This result indicates the good performance of the ET estimated based on the RTH model in this study.

## 4. Conclusions

An RTH model is proposed in this study and the RTH model is established based on the analysis of the relationship between PWV/T and ET residual. The initial value of ET for the RTH model is calculated using the TH model, and the time series of ET residual between TH and PM models are fitted based on multiple linear regression. An experiment is performed in the LP region, and 88 meteorological stations that are evenly distributed in the LP region over the period of 1979–2016 are selected. 31 GNSS stations derived from CMONOC located in this region are also used to analyze the performance of the RTH-based SPEI.

Comparison results show that the ET estimated based on the RTH model is superior to the traditional TH model, and the statistical result reveals that the average improvement rate of RMS derived from RTH-based ET is approximately 61.6% in the LP region. The RTH-based SPEI is validated and compared with the TH-based SPEI when the PM-based SPEI is regarded as a reference. The numerical result indicates the good performance of the RTH-based SPEI, whereas the quality of the TH-based SPEI is relatively poor at the selected meteorological stations. The statistical result reveals that the average improvement rate of the RMS derived from the RTH-based SPEI is approximately 48.0%, 49.3%, 43.2%, and 9.1% under 1-, 3-, 6-, and 12-month scales in the LP region, respectively. Finally, the RTH-based SPEI is tested in a case of April 2013 using the meteorological and GNSS stations. The results obtained above indicate the improved accuracy of the RTH-based SPEI, and further, verify the superiority of the proposed RTH model in this paper.

## Figures and Tables

**Table 1:** Statistical result of average RMS and MAE of TH- and RTH-based SPEI compared with the PM-based SPEI in the LP region over the period of 1979–2016.

Index	Model	Scale						
		1	3	6	12	18	24	Mean
RMS	TH	0.46	0.53	0.51	0.35	0.46	0.41	0.45
	RTH	0.24	0.27	0.29	0.35	0.36	0.41	0.32
MAE	TH	0.37	0.44	0.44	0.28	0.38	0.33	0.37
	RTH	0.20	0.22	0.23	0.28	0.30	0.33	0.26

## Appendix A

ET value can be calculated on the basis of the monthly mean temperature based on the empirical model proposed by [16], which is usually called the TH model and can be expressed as

(A1) $$\mathrm{ET}=\left\{ \begin{array}{ll} 0 & T\leq 0\\ 16K{\left(\frac{10T}{I}\right)}^{m} & T>0 \end{array}\right.$$

where T is the monthly mean temperature in °C; I is the heat index, which is calculated as the sum of 12 monthly index values i; i is calculated using the mean monthly temperature by the first formula in Equation (A2); m is a coefficient that is dependent on I, as described by the second formula in Equation (A2); and *K* is a correction coefficient computed as a function of the latitude and month, which can be expressed by the third formula in Equation (A2).

(A2) $$\begin{array}{l} i={\left(T/5\right)}^{1.514}\\ m=6.75\times 10^{-7}I^{3}-7.71\times 10^{-5}I^{2}+1.79\times 10^{-2}I+0.492\\ K=(N/12)\cdot (NDM/30) \end{array}$$

where *NDM* is the number of days of the month; and *N* is the maximum number of sun hours, which is usually obtained as follows:

(A3) $$N=(24/\pi )\cdot w_{s}$$

where $w_{s}$ is the hourly angle of the sun rising, which is calculated by

(A4) $$w_{s}=\arccos(-\tan\varphi \tan\delta )$$

where φ is the latitude in radians; and δ is the solar declination in radians, which can be obtained by

(A5) δ=0.4093sin(2πJ/365−1.405)

where *J* is the average Julian day of the month.

In addition to calculating the rough ET value based on the TH model, an accurate ETo value can be calculated using the PM model [17]. The PM model can be expressed as [47,48]:

(A6) $$ET=\frac{0.408\Delta (R_{n}-G)+\gamma \frac{900}{T+273.16}u_{2}(e_{s}-e_{a})}{\Delta +\gamma (1+0.34u_{2})}$$

where T and $u_{2}$ are the 2 m average air temperature in °C and wind speed in m s^−1^, respectively; $R_{n}$ is the crop surface net radiation in MJ m^−2^ d^−1^; *G* is the soil heat flux in MJ m^−2^ d^−1^; e^s^ is the saturation vapor pressure in kPa; $e_{a}$ is the actual vapor pressure in kPa; ∆ is the slope of the vapor pressure curve in kPa °C^−1^; γ is a psychrometric constant in kPa °C^−1^; 900 is the coefficient for the reference crop; and 0.34 is the wind coefficient for the reference crop. The preceding eight variables can be calculated using the daily maximum and minimum temperature, relative humidity, sunshine duration, and 2 m wind following the formulas of [41].

## Appendix B

SPEI can be calculated following the formula proposed by [5], where the monthly climatic water balance $D_{i}$ of month i is initially computed using the difference between precipitation $P_{i}$ and PET and expressed as follows:

(A7) $$D_{i}=P_{i}-ET_{i}$$

The calculated *D_i_* values are aggregated at different time scales. A good relationship exists between SPEI and the log-logistic distribution based on the Kolmogorov–Smirnov test under multi-month scale on a global scale [13], therefore, SPEI is calculated using the three-parameter log-logistic distribution based on the standardized *D* series. The probability distribution function of the log-logistic distribution for *D* series can be expressed as

(A8) $$F(x)={\left[1+{\left(\frac{\alpha }{x-\gamma }\right)}^{\beta }\right]}^{-1}$$

where α, β, and γ are the scale, shape, and origin parameters, respectively, which are obtained using the L-moment procedure [5].

(A9) $$\begin{array}{l} \beta =\frac{2w_{1}-w_{0}}{6w_{1}-w_{0}-w_{2}}\\ \alpha =\frac{(w0-2w1)\beta }{\Gamma (1+1/\beta )\Gamma (1-1/\beta )}\\ \gamma =w0-\alpha \Gamma (\frac{1+1}{\beta })\Gamma (\frac{1-1}{\beta }) \end{array}$$

where Γ(1+1/β) is the gamma function of (1+1/β), and $w_{s}$ is the probability-weighted moments of order *s* (*s* = 0, 1, 2) and can be calculated as follows:

(A10) $$w_{s}=\frac{1}{n}\sum_{n}^{n}{\left(1-\frac{j-0.35}{n}\right)}^{s}D_{i}$$

where *n* is the number of data points, and *j* is the range of observations in increasing order. SPEI is then calculated as the standardized values of F(x), as shown as follows:

(A11) $$\mathrm{SPEI}=W-\frac{2.515517+0.802853W+0.010328W^{2}}{1+1.432788W+0.189269W^{2}+0.001308W^{3}}$$

where $W=\sqrt{-2In(F(x))}$ when *F*(*x*) < 0.5, and $W=\sqrt{-2In(1-F(x))}$ when *F*(*x*) > 0.5. SPEI value of 0 represents 50% of the cumulative probability of *D* series.

## References

[1] Zhang Y., Peng C., Li W., Fang X., Zhang T., Zhu Q., Chen H., Zhao P. (2013). Monitoring and estimating drought-induced impacts on forest structure, growth, function, and ecosystem services using remote-sensing data: Recent progress and future challenges. Environ. Rev..

[2] Daryanto S., Wang L., Jacinthe P.-A. (2017). Global synthesis of drought effects on cereal, legume, tuber and root crops production: A review. Agric. Water Manag..

[3] Palmer W.C. (1965). Meteorological Drought.

[4] McKee T.B., Doesken N.J., Kleist J. (1993). The relationship of drought frequency and duration to time scales. Proceedings of the 8th Conference on Applied Climatology.

[5] Vicente-Serrano S.M., Beguería S., López-Moreno J.I. (2010). A multiscalar drought index sensitive to global warming: The standardized precipitation evapotranspiration index. J. Clim..

[6] Mishra A.K., Singh V.P. (2010). A review of drought concepts. J. Hydrol..

[7] Naumann G., Alfieri L., Wyser K., Mentaschi L., Betts R.A., Carrao H., Spinoni J., Vogt J., Feyen L. (2018). Global changes in drought conditions under different levels of warming. Geophys. Res. Lett..

[8] Guttman N.B. (1998). Comparing the palmer drought index and the standardized precipitation index1. JAWRA J. Am. Water Resour. Assoc..

[9] Redmond K.T. (2002). The depiction of drought: A commentary. Bull. Am. Meteorol. Soc..

[10] Zhang Q., Li J., Singh V.P., Bai Y. (2012). SPI-based evaluation of drought events in Xinjiang, China. Nat. Hazards.

[11] Zarch M.A.A., Sivakumar B., Sharma A. (2015). Droughts in a warming climate: A global assessment of Standardized precipitation index (SPI) and Reconnaissance drought index (RDI). J. Hydrol..

[12] Zhang Y., Wang J., Shen Z., Xie X. (2019). Evolution Characteristics of Seasonal Drought in Hunan Based on the Standardized Precipitation Index (SPI). Geoscience.

[13] Vicente-Serrano S.M., Beguería S., López-Moreno J.I., Angulo M., El Kenawy A. (2010). A new global 0.5 gridded dataset (1901–2006) of a multiscalar drought index: Comparison with current drought index datasets based on the Palmer Drought Severity Index. J. Hydrometeorol..

[14] Vicente-Serrano S.M., López-Moreno J.I., Lorenzo-Lacruz J., El Kenawy A., Azorin-Molina C., Morán-Tejeda E., Pasho E., Zabalza J., Beguería S., Angulo-Martínez M. (2011). The NAO impact on droughts in the Mediterranean region. Hydrological, Socioeconomic and Ecological Impacts of the North Atlantic Oscillation in the Mediterranean Region.

[15] Vicente S.S.M., Peña-Gallardo M., Hannaford J., Lorenzo-Lacruz J., Sbovoda M., Quiring S., Domínguez-Castro F., Maneta M., Tomas-Burguera M., Ahmed E.K. Climatic drought time-scales show varied spatial and seasonal effects on hydrological droughts in natural basins of US. Proceedings of the EGU General Assembly Conference Abstracts.

[16] Thornthwaite C.W. (1948). An approach toward a rational classification of climate. Geogr. Rev..

[17] Allen R.G., Pereira L.S., Raes D., Smith M. (1998). Crop Evapotranspiration-Guidelines for Computing Crop Water Requirements-FAO Irrigation and Drainage Paper 56.

[18] Sheffield J., Wood E.F., Roderick M.L. (2012). Little change in global drought over the past 60 years. Nature.

[19] Bevis M., Businger S., Herring T.A., Rocken C., Anthes R.A., Ware R.H. (1992). GPS meteorology: Remote sensing of atmospheric water vapor using the Global Positioning System. J. Geophys. Res. Atmos..

[20] Duan J., Bevis M., Fang P., Bock Y., Chiswell S., Businger S., Rocken C., Solheim F., van Hove T., Ware R. (1996). GPS meteorology: Direct estimation of the absolute value of precipitable water. J. Appl. Meteorol..

[21] Niell A.E. (2001). Preliminary evaluation of atmospheric mapping functions based on numerical weather models. Phys. Chem. Earth Part A Solid Earth Geod..

[22] Memmo A., Fionda E., Paolucci T., Cimini D., Ferretti R., Bonafoni S., Ciotti P. (2005). Comparison of MM5 integrated water vapor with microwave radiometer, GPS, and radiosonde measurements. IEEE Trans. Geosci. Remote Sens..

[23] Lu C., Li X., Li Z., Heinkelmann R., Nilsson T., Dick G., Ge M., Schuh H. (2016). GNSS tropospheric gradients with high temporal resolution and their effect on precise positioning. J. Geophys. Res. Atmos..

[24] Zhao Q., Yao Y., Yao W., Zhang S. (2019). GNSS-derived PWV and comparison with radiosonde and ECMWF ERA-Interim data over mainland China. J. Atmos. Sol. Terr. Phys..

[25] Huelsing H.K., Wang J., Mears C., Braun J.J. (2017). Precipitable water characteristics during the 2013 Colorado flood using ground-based GPS measurements. Atmos. Meas. Tech..

[26] Zhao Q., Yao Y., Yao W., Li Z. (2018). Real-time precise point positioning-based zenith tropospheric delay for precipitation forecasting. Sci. Rep..

[27] Muhammad M., Abdullah M., Singh M.J., Suparta W., Islam M.T., Tangang F. Characterization of GPS PWV during flooding event over Keningau, Sabah. Proceedings of the 2013 IEEE International Conference on Space Science and Communication (IconSpace).

[28] Choy S., Wang C., Zhang K., Kuleshov Y. (2013). GPS sensing of precipitable water vapour during the March 2010 Melbourne storm. Adv. Space Res..

[29] Jiang W., Yuan P., Chen H., Cai J., Li Z., Chao N., Sneeuw N. (2017). Annual variations of monsoon and drought detected by GPS: A case study in Yunnan, China. Sci. Rep..

[30] Wang X., Zhang K., Wu S., Li Z., Cheng Y., Li L., Yuan H. (2018). The correlation between GNSS-derived precipitable water vapor and sea surface temperature and its responses to El Niño–Southern Oscillation. Remote Sens. Environ..

[31] Yang Y., Bian Y. Building a harmonious relationship between water resource and the environment on the Loess Plateau: How to restore its vegetation. Proceedings of the 2011 International Symposium on Water Resource and Environmental Protection.

[32] Gao X., Zhao Q., Zhao X., Wu P., Pan W., Gao X., Sun M. (2017). Temporal and spatial evolution of the standardized precipitation evapotranspiration index (SPEI) in the Loess Plateau under climate change from 2001 to 2050. Sci. Total Environ..

[33] Li Z., Zheng F.L., Liu W.Z., Jiang D.J. (2012). Spatially downscaling GCMs outputs to project changes in extreme precipitation and temperature events on the Loess Plateau of China during the 21st Century. Glob. Planet. Chang..

[34] Gui K., Che H., Chen Q., Zeng Z., Liu H., Wang Y., Zheng Y., Sun T., Liao T., Wang H. (2017). Evaluation of radiosonde, MODIS-NIR-Clear, and AERONET precipitable water vapor using IGS ground-based GPS measurements over China. Atmos. Res..

[35] Kouba J. (2009). A Guide to Using International GNSS Service (IGS) Products.

[36] Perler D., Geiger A., Hurter F. (2011). 4D GPS water vapor tomography: New parameterized approaches. J. Geod..

[37] Saastamoinen J. (1972). Atmospheric correction for the troposphere and stratosphere in radio ranging satellites. Use Artif. Satell. Geod..

[38] Askne J., Nordius H. (1987). Estimation of tropospheric delay for microwaves from surface weather data. Radio Sci..

[39] Bevis M., Businger S., Chiswell S., Herring T.A., Anthes R.A., Rocken C., Ware R.H. (1994). GPS meteorology: Mapping zenith wet delays onto precipitable water. J. Appl. Meteorol..

[40] Ding M. (2018). A neural network model for predicting weighted mean temperature. J. Geod..

[41] Yang K., He J., Tang W., Qin J., Cheng C.C. (2010). On downward shortwave and longwave radiations over high altitude regions: Observation and modeling in the Tibetan Plateau. Agric. For. Meteorol..

[42] Chen Y., Yang K., He J., Qin J., Shi J., Du J., He Q. (2011). Improving land surface temperature modeling for dry land of China. J. Geophys. Res.

[43] Li J., Sun X. (2015). Evaluation of changes of Thornthwaite Moisture Index in Victoria. Aust. Geomech. J..

[44] Karunarathne A.M.A.N., Gad E.F., Disfani M.M., Sivanerupan S., Wilson J.L. (2016). Review of calculation procedures of Thornthwaite Moisture Index and its impact on footing design. Aust. Geomech. J..

[45] Zhang W., Lou Y., Haase J.S., Zhang R., Zheng G., Huang J., Shi C., Liu J. (2017). The use of ground-based GPS precipitable water measurements over China to assess radiosonde and ERA-Interim moisture trends and errors from 1999 to 2015. J. Clim..

[46] Wang X., Cheng Y., Wu S., Zhang K. (2016). An effective toolkit for the interpolation and gross error detection of GPS time series. Surv. Rev..

[47] Allen R.G. (1994). An update for the calculation of reference evapotranspiration. ICID Bull..

[48] Allen R.G., Smith M., Perrier A., Pereira L.S. (1994). An update for the definition of reference evapotranspiration. ICID Bull..

